# A Novel Index Using Ankle Hemodynamic Parameters to Assess the Severity of Peripheral Arterial Disease: A Pilot Study

**DOI:** 10.1371/journal.pone.0164756

**Published:** 2016-10-19

**Authors:** Jun Tanno, Yodo Gatate, Takatoshi Kasai, Shintaro Nakano, Takaaki Senbonmatsu, Osamu Sato, Shigeru Ichioka, Makoto Kuro-o, Shigeyuki Nishimura

**Affiliations:** 1 Department of Cardiology, Saitama Medical University, International Medical Center, Saitama, Japan; 2 Cardiovascular Respiratory Sleep Medicine, Department of Cardiovascular Medicine, Juntendo University Graduate School of Medicine, Tokyo, Japan; 3 Department of Vascular Surgery Saitama Medical Center, Saitama Medical University, Saitama, Japan; 4 Department of Plastic and Reconstructive Surgery, Saitama Medical University, Saitama, Japan; 5 Division of Anti-Aging Medicine, Center for Molecular Medicine, Jichi Medical University, Tochigi, Japan; The University of Tokyo, JAPAN

## Abstract

In peripheral arterial disease (PAD) of the lower extremities, the presence of flow-limiting stenoses can be objectively detected by the ankle-brachial index (ABI). However, the severity of ischemic symptoms is not necessarily associated with the ABI value. Atherosclerotic plaque in lower extremity PAD induces ankle arterial stiffness and reduces ankle vascular resistance, which may decrease ankle blood flow and cause ischemic symptoms. We hypothesized that the ankle hemodynamic index (AHI), defined as the ratio of ankle arterial stiffness to ankle vascular resistance, could be used to assess the blood supply deficiency in a diseased lower limb in patients with PAD. The 85 consecutive patients with PAD who were retrospectively analyzed in this study had Rutherford grade 1 to grade 6 ischemia diagnosed as PAD and significant stenotic lesions (>50% diameter stenosis) of the lower extremity on contrast angiography. The AHI was calculated as the product of the ankle pulse pressure and the ratio of heart rate to ankle mean arterial pressure (ankle pulse pressure × heart rate/ankle mean arterial pressure). The Rutherford grade was significantly correlated with the AHI (*r* = 0.50, *P* < 0.001), but not with the ABI (*r* = 0.07, *P* = 0.52). Multiple ordinal regression analysis showed that anemia (odds ratio 0.66, *P* = 0.002) and AHI (odds ratio 1.04, *P* = 0.02) were independently associated with Rutherford grade. Our study shows that AHI, a novel parameter based on the ABI measurement, is well correlated with ischemic symptoms, and may be a useful means to assess the arterial blood supply of the lower extremities of patients with PAD.

## Introduction

Peripheral arterial disease (PAD) is a significant public health concern; worldwide more than 202 million adults have been diagnosed with the disease. Moreover, it poses a significant economic burden, reflecting the treatment of advanced disease, the alleviation of symptoms, and the prevention and treatment of ischemic events [1, 2]. PAD of the lower extremities, in which the blood supply is obstructed mainly by atherosclerosis [3], ranges in severity from claudication to critical limb ischemia with tissue loss. In PAD, atherosclerotic plaque encroaches on the peripheral artery lumen, decreasing the blood flow and vascular reactivity of the arteries [4, 5]. The severity of lower limb ischemia among patients with PAD is most commonly classified according to the Rutherford and Fontaine classification systems, both of which are symptom-based [6–8]. The Rutherford classification system has also been used to determine the revascularization potential of lower extremities with ischemia [8, 9].

The presence of flow-limiting stenoses can also be objectively and noninvasively detected using the ankle-brachial index (ABI), defined as the ratio of systolic blood pressures in the ankle artery to those in the brachial artery, to assess the hemodynamics of the lower limbs [7]. In fact, the ABI is the main clinical measure of the presence and severity of PAD [10]. An ABI of <0.9 confirms the diagnosis of PAD, while an ABI of <0.4 indicates critical limb ischemia [7, 8]. However, the severity of ischemic symptoms is not necessarily associated with the ABI value for the following reasons. First, patients with either severely stenotic or totally occluded iliofemoral arteries may have a normal ABI at rest if sufficient collaterals are present [4, 8, 11]. Second, in patients with diabetes mellitus and in the elderly, the ABI may be high if non-compressive arteries are present [9, 12]. Thus, in the clinical setting there are few methods that allow an objective and noninvasive assessment of a deficient blood supply in the diseased limbs of patients with PAD.

**Fig 1:**
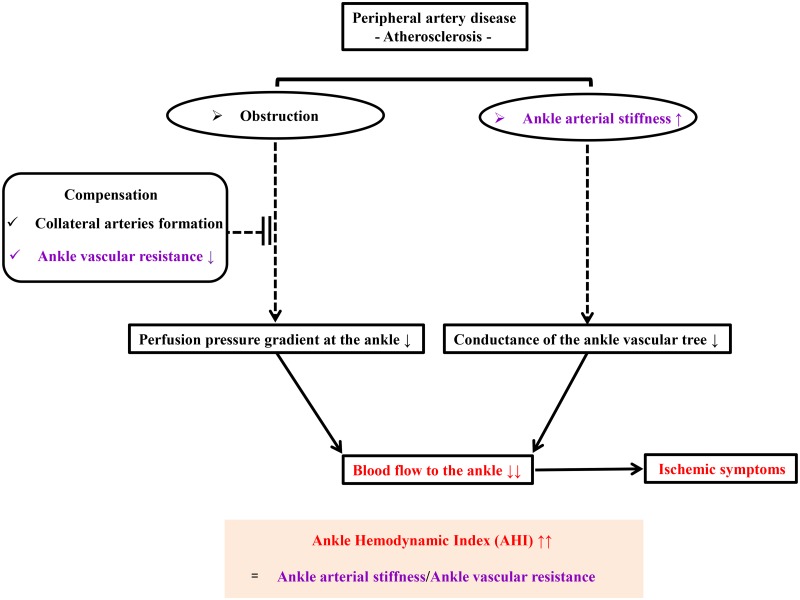
A flow-chart of the concepts of the AHI. AHI, ankle hemodynamic index.

Blood flow to the lower extremities or ankles reflects the difference between arterial and venous pressures (perfusion pressure gradient) and the conductance of the vascular tree [13]. In patients with PAD, the blood supply (blood flow) to the diseased limb may be reduced by: 1) a decrease in the ankle perfusion pressure gradient due to arterial obstruction, which may be partially compensated by collateral arteries or ankle vascular resistance [4, 5]; or 2) a decrease in the conductance of the ankle vascular trees (ankle vascular compliance) secondary to calcified, poorly compressible arteries (ankle arterial stiffness) [9]. Ankle vascular resistance is the resistance to ankle blood flow of the ankle vasculature, which can be expressed as the mean arterial pressure (MAP) at the ankle/α × cardiac output (stroke volume (SV) × heart rate) where α is a proportionality coefficient, i.e., the blood volume at the ankle per minute. Ankle vascular compliance is the change in volume (ΔV) at the ankle divided by the change in pressure (ΔP) at the ankle [14, 15], which can be expressed as α × SV/ankle pulse pressure (PP) (α × SV/ankle PP), as SV represents the change in blood volume at the ankle per one heartbeat. Therefore, ankle arterial stiffness, which is the reciprocal of the product of ankle vascular compliance, can be expressed as ankle PP/(α × SV). Taken together, the above-mentioned two indicators, the decreased vascular resistance (ankle MAP/(α × SV) × heart rate) and the increased ankle arterial stiffness (ankle PP/(α × SV)) may play an essential role in the poor blood supply to the diseased limb. Thus, we suppose that the increased ankle hemodynamic index (AHI), defined as the ratio of the ankle arterial stiffness to the vascular resistance (ankle PP × heart rate/ankle MAP), may augment the clinical importance in these patients, rather than ABI (Fig 1).

We hypothesized that the AHI, an easily obtainable and novel parameter based on ABI measurements, may be correlated and associated with ischemic symptoms in patients with PAD, and could be used to objectively and noninvasively assess deficient blood supply in diseased limbs. The purpose of this study was to investigate the correlation compared with ABI and the association between AHI and the severity of lower limb ischemia, classified by the Rutherford grade. Thus, we aimed to: 1) evaluate the correlation between the AHI, ABI, and the Rutherford grade, and 2) to determine the independent variables associated with the Rutherford grade using AHI and other clinical variables such as patients’ characteristics and objective clinical variables.

## Methods

### Ethics statement

The clinical investigation was conducted according to the principles stated in the Declaration of Helsinki. The study protocol was approved by the Institutional Review Board of Saitama Medical University International Medical Center. Written informed consent for the examination, including angiography, was obtained from all patients.

### Study population

The 115 patients consecutively enrolled in this study between May 2011 and February 2015 were evaluated for Rutherford classification (grades 1–6) [6, 7] by their cardiologist or dermatologist. PAD was diagnosed in those with significant stenotic lesions (>50% stenosis on visual estimation) of the lower extremity as seen on contrast angiography [7, 8]. Thirty patients were excluded because of bilateral symptoms (n = 10), acute limb ischemia (n = 3), no ABI data (n = 10), and immeasurable ankle blood pressure (n = 7).

### Measurement and definition of ABI and AHI

Before the contrast angiography examination, patients were evaluated for ABI and AHI after resting for 5 min in the supine position. Measurements were made using a previously validated [8, 16] automated oscillometric device (VaSera^™^; Fukuda Dehnshi Co., Ltd., Tokyo, Japan). ABI and AHI were calculated based on bilateral brachial systolic blood pressure (SBP), bilateral ankle SBP, ankle diastolic blood pressure (DBP), ankle PP, ankle MAP, and heart rate, which were measured simultaneously during ABI determination. ABI was defined as the ankle SBP of the diseased limb/the highest SBP of that measured in each arm [8, 17], and AHI as the ankle PP × heart rate/ankle MAP of the diseased limb, as described in the Introduction.

### Data collection

Baseline patient data, including age, sex, height, weight, and body mass index (BMI), were recorded. Hypertension was defined as current or previous treatment with antihypertensive medication, diabetes as current or previous treatment with antidiabetic medication (insulin or oral hypoglycemic drugs), and dyslipidemia as current or previous treatment with antidyslipidemic medication. A history of either end-stage renal disease requiring hemodialysis or collagen disease (rheumatoid arthritis, systemic sclerosis, mixed connective tissue disease) was recorded based on interviews with the patients and/or their relatives. Medications including aspirin, clopidogrel, cilostazol, warfarin, statins, and angiotensin-converting enzyme (ACE) inhibitors at the time of ABI measurement were also recorded. Laboratory data consisted of hemoglobin, creatinine, and C-reactive protein (CRP) levels, activated partial thromboplastin time (APTT), prothrombin time-international normalized ratio (PT-INR), and albumin. Left ventricular ejection fraction, measured by transthoracic echocardiography, was also recorded.

### Statistical analyses

All statistical analyses were performed using SPSS statistical software (version 18.0; SPSS Inc., Chicago, IL, USA). Continuous variables are expressed as the mean±standard deviation or median (first to third quartile), and categorical variables as numbers and percentages. The normality of continuous variables was assessed using the Shapiro–Wilk test. The relationship between ABI or AHI and Rutherford grade was analyzed using Spearman’s correlation coefficient. Associations between clinical variables, including AHI, and Rutherford grade, were analyzed using univariate and multivariate ordinal logistic regression and expressed as odds ratio (OR) with 95% confidence interval. The univariate ordinal regression analysis for factors related to Rutherford grade included age, sex, height, weight, BMI, presence of diabetes, hypertension, or dyslipidemia, current smoking, history of hemodialysis or collagen disease, higher brachial SBP, heart rate, ejection fraction, hemoglobin level, creatinine, CRP, and APTT levels, PT-INR, routine use of low-dose aspirin, clopidogrel, cilostazol, warfarin, statin, or ACE inhibitors, and ankle SBP, DBP, PP, MAP, ABI, and AHI in the diseased limb. Independent baseline variables with a *P* value <0.05 in the univariate analyses were included in the multivariate analyses; collinearity was verified for each pair of variables. Because higher brachial SBP, heart rate, ankle PP, and ankle MAP were components of both the AHI and the ABI, they were excluded from the multiple ordinal logistic regression model to allow multiple collinearities to be taken into account. A *P* value of <0.05 was considered to indicate statistical significance for all analyses.

## Results

### Patient characteristics, medical therapy, and Rutherford grade

Significant stenotic lesions were detected on contrast angiography in the diseased limbs (n = 85) of the 85 patients (60 males and 25 females). Patient characteristics, medical therapy, and Rutherford grade are shown in Table 1. Most patients were elderly, non-obese men with preserved left ventricular systolic function. Among the 85 patients, 54 (63.5%) had diabetes, 65 (76.5%) had hypertension, and 40 (47.1%) had dyslipidemia. A history of hemodialysis and collagen disease was observed in 22 (25.9%) and 10 (11.8%) patients, respectively. At the ABI measurement, the median higher brachial SPB was 146 mmHg (first–third quartile: 133–158 mmHg) and heart rate was 71.1 ± 13.2 beats/min (range 47–112 beats/min). At the ABI measurement, mean hemoglobin level was 12.3 ± 2.0 g/dl (range 7.1–16.1 g/dl), median creatinine level was 1.06 mg/dl (first–third quartile: 0.813–3.49 mg/dl), and median CRP was 0.35 mg/dl (first–third quartile: 0.08–1.55 mg/dl). Antiplatelet agents (low-dose aspirin, clopidogrel, cilostazol) or warfarin were used by 62 (72.9%) patients, statins by 30 (35.3%), and ACE inhibitors by 7 (8.2%) patients. A totally occluded iliofemoral artery was detected in 33 (36.7%) patients. The median Rutherford grade was 3 (first–third quartile: 3–6); 8 (9.4%) patients had Rutherford grade 1, 13 (15.3%) grade 2, 23 (27.1%) grade 3, 4 (4.7%) grade 4, 15 (17.6%) grade 5, and 22 (25.9%) grade 6.

**Table 1:** Patient characteristics, medical therapy, and Rutherford grade.

Demographics	
Mean age, years	72.0 (65.0–76.0)
Male, %	70.6 (60/85)
Weight, kg	57.6 ± 11.6
Height, cm	159.9 ± 9.9
BMI, kg/m^2^	22.6 ± 3.8
Coexisting conditions	
Diabetes mellitus, %	63.5 (54/85)
Hypertension, %	76.5 (65/85)
Dyslipidemia, %	47.1 (40/85)
Current smoker, %	21.2 (18/85)
Regular hemodialysis, %	25.9 (22/85)
Collagen disease, %	11.8 (10/85)
Findings at ABI measurement	
Higher brachial SBP, mmHg	146 (133–158)
Heart rate, beats/min	71.1 ± 13.2
EF, %	59.6 ± 14.3
Hb, g/dl	12.3 ± 2.0
Cr, mg/dl	1.06 (0.81–3.49)
CRP, mg/dl	0.35 (0.08–1.55)
APTT, sec.	31.4 (29.3–35.3)
PT-INR	1.05 (1.01–1.14)
Medication	
Antiplatelet agent or warfarin, %	72.9 (62/85)
Low dose aspirin, %	49.4 (42/85)
Clopidogrel, %	23.5 (20/85)
Cilostazol, %	20.0 (17/85)
Warfarin, %	12.9 (11/85)
Statin, %	35.3 (30/85)
ACE inhibitor, %	8.2 (7/85)
Contrast angiography findings	
Totally occluded iliofemoral artery, %	37.6 (33/85)
Rutherford grade	3 (3–6)
1, %	9.4% (8/85)
2, %	15.3% (13/85)
3, %	27.1% (23/85)
4, %	4.7% (4/85)
5, %	17.6% (15/85)
6, %	25.9% (22/85)

BMI, body mass index; ABI, ankle-brachial index; SBP, systolic blood pressure; EF, ejection fraction; Hb, hemoglobin; Cr, creatinine; CRP, C-reactive protein; APTT, activated partial thromboplastin time; PT-INR, prothrombin time-international normalized ratio; ACE, angiotensin-converting enzyme.

### Ankle blood pressure parameters, ABI, and AHI obtained during ABI measurement

All ankle blood pressure parameters, including AHI, were obtained by measuring the ABI in the diseased lower limb of each patient. They are shown in Table 2.

**Table 2:** Ankle blood pressure parameters, ABI, and AHI (derived from the ABI measurement).

ABI	0.69 ± 0.16
Ankle SBP, mmHg	99.9 ± 26.3
Ankle DBP, mmHg	60.9 ± 15.9
Ankle pulse pressure, mmHg	33.0 (22.0–54.0)
Ankle MAP, mmHg	79.0 ± 18.6
AHI	30.4 (21.0–46.9)

ABI, ankle-brachial index; SBP, systolic blood pressure; DBP, diastolic blood pressure; PP, pulse pressure; MAP, mean arterial pressure; AHI, ankle hemodynamic index.

### Correlations between Rutherford grade and ABI or AHI

**Fig 2:**
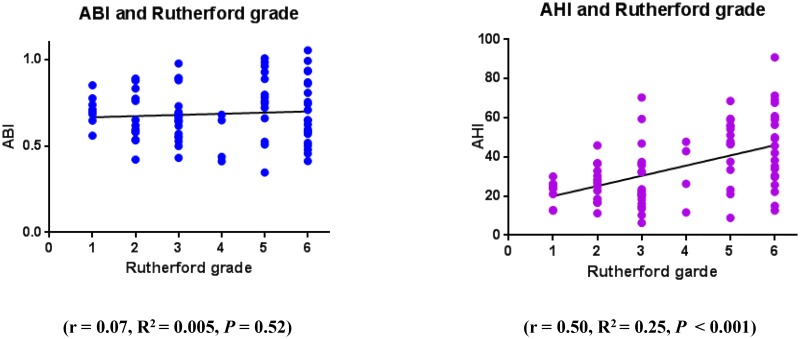
Correlation between the Rutherford classification and ABI and AHI. The correlation between Rutherford grade and AHI (*r* = 0.50, *R*^*2*^ = 0.25, *P* < 0.001) but not between Rutherford grade and ABI (*r* = 0.07, *R*^*2*^ = 0.005, *P* = 0.52) was significant. ABI, ankle-brachial index; AHI, ankle hemodynamic index.

As shown in Fig 2, the correlation between Rutherford grade and AHI (*r* = 0.50, *R*^*2*^ = 0.25, *P* < 0.001) but not between Rutherford grade and ABI (*r* = 0.07, *R*^*2*^ = 0.005, *P* = 0.52) was significant.

**Fig 3:**
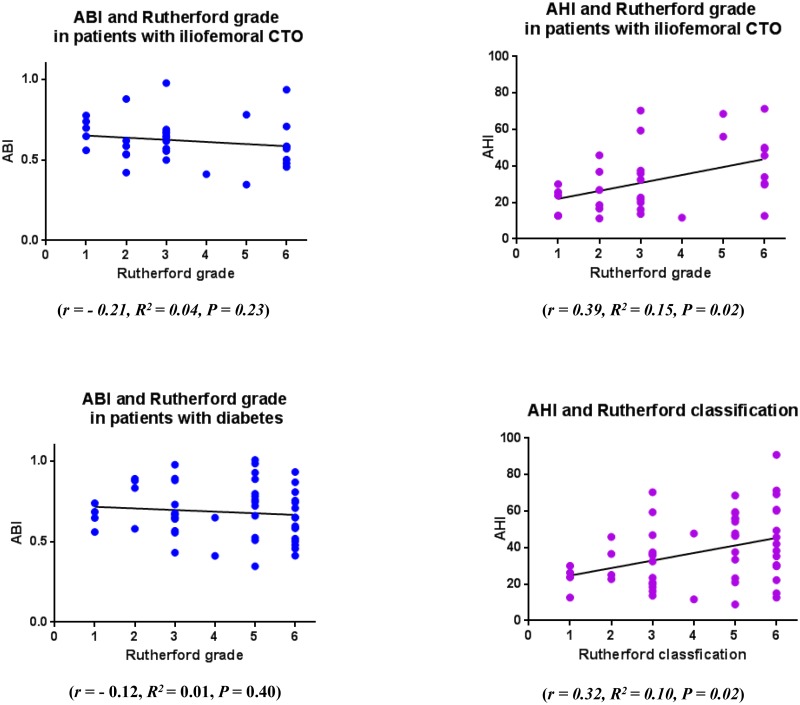
Correlation between the Rutherford classification and ABI and AHI in patients with a totally occluded iliofemoral artery or diabetes. In the subset of patients with totally occluded iliofemoral arteries (n = 33) or those with diabetes (n = 54) the correlation between Rutherford grade and AHI (*r* = 0.39, *R*^*2*^ = 0.15, *P =* 0.02 and *r* = 0.32, *R*^*2*^ = 0.10, *P =* 0.02, respectively) but not between Rutherford grade and ABI (*r* = −0.21, *R*^*2*^ = 0.04, *P* = 0.23, *r* = −0.12, *R*^*2*^ = 0.01, *P* = 0.40) was significant. ABI, ankle-brachial index; AHI, ankle hemodynamic index.

In the subset of patients with totally occluded iliofemoral arteries (n = 33) or those with diabetes (n = 54), ABI did not correlate significantly with Rutherford grade (totally occluded iliofemoral arteries: *r* = −0.21, *R*^*2*^ = 0.04, *P = 0*.*23*, and diabetes: *r* = −0.12, R^2^ = 0.01, *P* = 0.40, respectively). However, in both groups, Rutherford grade correlated significantly with AHI (totally occluded iliofemoral arteries: *r* = 0.39, *R*^*2*^ = 0.15, *P* = 0.02 and diabetes: *r* = 0.32, *R*^*2*^ = 0.10, *P* = 0.02; Fig 3).

### Associations between Rutherford grade and independent variables including AHI

Univariate analyses showed that diabetes (OR = 3.19, *P* = 0.005), regular hemodialysis (OR = 6.46, *P* < 0.001), higher heart rate (OR = 1.04, *P* = 0.02), lower hemoglobin level (OR = 0.53, *P* < 0.001), higher creatinine level (OR = 1.27, *P =* 0.001), higher CRP level (OR = 1.67, *P =* 0.001), absence of statin treatment (OR = 0.37, *P =* 0.02), lower ankle DBP (OR = 0.97, *P =* 0.007), higher ankle PP (OR = 1.03, *P =* 0.001), and higher AHI (OR = 1.06, *P* < 0.001) were significantly associated with a higher Rutherford grade (Table 3).

**Table 3:** Univariate analyses of relationships between Rutherford grade and independent variables.

	OR	95%CI	*P*-value
Age	0.99	0.96–1.03	0.74
Male	0.47	0.20–1.10	0.08
Height	0.99	0.96–1.03	0.70
Weight	0.99	0.96–1.02	0.42
BMI	1.00	0.90–1.10	0.94
Diabetes mellitus	0.99	1.41–7.21	0.005
Hypertension	1.00	0.66–3.92	0.30
Dyslipidemia	0.79	0.37–1.67	0.53
Current smoker	0.62	0.25–1.65	0.31
Regular hemodialysis	6.46	2.48–16.84	<0.001
Collagen disease	2.64	0.78–8.86	0.12
Higher brachial SBP	1.02	1.00–1.03	0.08
Heart rate	1.04	1.01–1.07	0.02
EF	0.99	0.97–1.02	0.67
Hb	0.53	0.42–0.66	<0.001
Cr	1.27	1.10–1.47	0.001
CRP	1.67	1.27–2.20	<0.001
APTT	1.58	0.99–1.11	0.11
PT–INR	1.45	0.62–3.36	0.39
Low dose aspirin	0.78	0.36–1.65	0.51
Clopidogrel	1.03	0.42–2.51	0.95
Cilostazol	1.34	0.52–3.44	0.55
Warfarin	1.04	0.34–3.21	0.94
Statin	0.37	0.17–0.84	0.02
ACE inhibitor	0.42	0.10–1.66	0.21
Ankle SBP	1.01	1.00–1.03	0.18
Ankle DBP	0.97	0.94–0.99	0.007
Ankle PP	1.03	1.02–1.05	0.001
Ankle MAP	1.01	0.99–1.03	0.59
ABI	1.92	0.18–20.21	0.59
AHI	1.06	1.03–1.08	<0.001

CI, confidence interval; BMI, body mass index; SBP, systolic blood pressure; EF, ejection fraction; Hb, hemoglobin; Cr, creatinine; CRP, C-reactive protein; APTT, activated partial thromboplastin time; PT-INR, prothrombin time-international; ACE, angiotensin-converting enzyme; DBP, diastolic blood pressure; PP, pulse pressure; MAP, mean arterial pressure; ABI, ankle-brachial index; AHI, ankle hemodynamic index.

Multivariate analysis showed that a higher CRP level (OR = 1.26, *P =* 0.09) was marginally associated with a higher Rutherford grade, whereas the association between a lower hemoglobin level (OR = 0.66, *P =* 0.002) and higher AHI (OR = 1.04, *P =* 0.02) and a higher Rutherford grade were significant (Table 4).

**Table 4:** Multiple ordinal regression analysis of the relationships between Rutherford grade and independent variables.

Independent variable	OR	95%CI	*P*-value
Diabetes mellitus	1.44	0.61–3.42	0.41
Regular hemodialysis	0.74	0.07–8.45	0.81
Hb	0.66	0.51–0.86	0.002
Cr	1.13	0.81–1.57	0.48
CRP	1.26	0.96–1.65	0.09
Statin	0.52	0.21–1.29	0.16
Ankle DBP	1.00	0.97–1.03	0.97
AHI	1.04	1.01–1.05	0.02

CI, confidence interval; Hb, hemoglobin; Cr, creatinine; CRP, C-reactive protein; DBP, diastolic blood pressure; AHI, ankle hemodynamic index.

## Discussion

The major findings of the present study are: 1) AHI, but not ABI, is significantly correlated with the Rutherford grade (the higher the AHI, the higher the Rutherford grade) and 2) anemia and increased AHI are independently associated with a higher Rutherford grade, as determined in a multiple ordinal regression analysis. These findings suggest that the AHI may objectively provide the severity of the blood supply limitation among PAD patients, and can be a useful supportive means to assess their symptoms, especially in those whose subjective symptoms are unreliable.

### The role of AHI compared with ABI in PAD

Both the steady (i.e., MAP) and the pulsatile (i.e., SBP, DBP, and PP) components of arterial pressure [18] should be comprehensively integrated in the assessment of the diseased limb/s of patients with PAD, primarily because the hemodynamics of PAD impair two principal functions of the arterial system, namely the conduit (corresponding to a steady component) and the cushion (corresponding to a pulsatile component) functions [19]. The AHI implies both steady and pulsatile arterial components.

The conduit function (i.e., vascular resistance) where blood from the heart is delivered to capillaries of organs and tissues according to their need [19], is steadily dependent on MAP [15]. The cushion function (i.e., arterial stiffness), where pulsations from the heart are cushioned by the arteries to allow constant capillary blood flow, is dependent on the PP [15]. Interestingly, HR, which also constitutes AHI, plays an essential role in regulating both steady and pulsatile arterial components [15]. Indeed, compared with the ABI, which only implies the pulsatile component of arterial function, AHI, which implies the steady as well as the pulsatile component and HR, demonstrated a significant association with the degree of lower limb ischemia as assessed by the Rutherford classification in our study.

Thus, the AHI, which reflects both the pulsatile (i.e., PP) and steady (i.e., MAP) components, more closely reflects the degree of lower limb ischemia as assessed by the Rutherford classification. Based on a definition of the AHI as ankle arterial stiffness/ankle vascular resistance, both components, i.e., advanced arterial stiffness of the ankle and a decrease in ankle vascular resistance, including compensation by the collateral circulation, may result in an increased AHI. We suspect that the formation of collateral circulation or decreased vascular resistance may play a protective role to facilitate the blood supply in early stages; however, in cases with advanced ischemia, these systems may not satisfactorily afford blood supply to ischemic limbs. Therefore, AHI may also reliably predict a deficiency in the arterial blood supply. Indeed, in our study, there was a significant correlation between AHI and Rutherford grade in all 85 patients and the subgroup with a totally occluded iliofemoral artery or a history of diabetes.

In contrast, the ABI, which reflects only the pulsatile component (i.e., SBP), does not necessarily reflect the degree of lower limb ischemia determined using the Rutherford classification. Thus, in patients with advanced ankle arterial stiffness due to arterial calcification or a decrease in arterial vascular resistance secondary to compensation by collateral flow [20], an underestimation or an overestimation for ischemia determined by ABI may occur. In our study, the ABI was not correlated with the Rutherford grade either in all patients, or in the subgroup with a totally occluded iliofemoral artery or diabetes.

### The role of independent factors associated with Rutherford grade

The Rutherford classification is used to assess the severity of lower limb ischemia in patients with PAD, in which arterial stenosis, occlusion, and calcified arteries are related to atherosclerosis [7, 12]. Arterial damage decreases the arterial blood supply to the lower extremities, both during exercise and at rest, despite compensation by collateral flow and ankle vascular resistance [12, 21], and causes symptoms ranging from intermittent claudication to ischemic skin lesions. Therefore, the Rutherford classification, a clinical symptom-based measure to evaluate ischemic limb, may reflect the influence of multiple factors such as atherosclerotic obstructions, arterial calcification, or compensatory adaptation. Indeed, we found that anemia, higher CRP level, and higher AHI were significantly associated with a higher Rutherford grade.

Red blood cells (RBCs) and circulating hemoglobin mediate physiological processes, including oxygen carrying capacity, but are also involved in pathophysiological pathways, such as inflammatory processes, oxidative stress, and blood viscosity disturbances [22]. Because both RBCs and hemoglobin contribute to regulating functional capacity in patients with PAD [12], anemia will directly exacerbate their lower limb ischemia [12, 22]. In our study, anemia was one of the independent correlates of Rutherford grade. The CRP level may also be associated with atherosclerosis and its progression [23]; in our study, it was marginally associated with the Rutherford grade. Therefore, anemia and the CRP level may be of value as indirect indicators of the lower limb ischemia that accompanies PAD. AHI, by representing hemodynamic components such as SBP, DBP, and heart rate, can also predict the severity of lower limb ischemia evaluated by the Rutherford classification. In this study, AHI was an independent correlate of the Rutherford grade. Thus, in contrast to anemia and CRP level, the AHI offers a direct measure of the blood supply deficiency in patients with PAD.

### Clinical implications

Measuring blood pressure in the arteries of the ankle has become a standard part of the initial evaluation of the severity of the atherosclerotic burden in patients with PAD [7, 8]. The findings of this study suggest that the AHI, calculated from the parameters obtained during measurement of ABI, can be used to objectively and noninvasively assess the degree of arterial blood supply deficiency in the diseased limbs, and may support the subjective assessment. Additionally, it may also be useful to easily and objectively determine the revascularization potential of lower extremities with ischemia and to assess the degree of response to treatments (i.e. medical therapy, exercise therapy, revascularization).

### Limitations

This study has several limitations. First, the number of patients was limited because only those presenting with significant (>50%) stenosis in the lower extremity on contrast angiography and those with measurable ankle blood pressure were included. However, the number of included patients was sufficient to show a clear relationship between AHI and Rutherford grade. Second, the ABI was measured by oscillometric method, not by Doppler ultrasound method. However, according to the scientific statement from the American Heart Association, oscillometric method is also primarily used to detect limb flow and pulse volume for measuring the ABI [17]. In addition, Beckman et al. demonstrated that in the subgroup of patients with PAD, the ABI measured by oscillometric method correlated well with that measured by Doppler method [24]. Finally, this is an observational, cross-sectional study, and the AHI was measured by oscillometric device at rest. The predictive value of the AHI or its relationship with stress test-based evaluation of ischemia has not been studied. Further studies, including comparing skin perfusion pressure and AHI are required to address the unknown roles of the AHI.

### Conclusions

This study identified a significant correlation between the AHI, but not the ABI, and the Rutherford grade. Multiple ordinal regression analysis showed a significant and independent association of the AHI with the Rutherford classification. Our findings suggest that AHI, a novel parameter based on the ABI measurement, can be used to assess the arterial blood supply in the lower extremities of patients with PAD.

## Supporting Information

S1 File Individual patient-level data underlying results presented in the tables of this study. (PDF)
