# Supplementary material for: A Novel Index Using Ankle Hemodynamic Parameters to Assess the Severity of Peripheral Arterial Disease: A Pilot Study
Source: PLoS One. 2016 Oct 19;11(10):e0164756. doi: 10.1371/journal.pone.0164756 (PMC5070739; doi:10.1371/journal.pone.0164756)
Supplement: S1 File — (PDF) [file pone.0164756.s001.pdf]

Patients No.	gender（m:0, f:1）	age	height	body weight
1	0	68	156	52
2	0	65	165	53
3	0	83	145	40
4	0	75	157	52
5	0	64	169	58
6	0	74	177	74
7	0	75	161	68
8	0	56	168	77
9	1	80	148	41
10	0	66	158	50
11	0	72	168	60.7
12	0	69	161	61
13	1	77	154	49
14	0	70	162	67
15	0	76	162	45
16	1	65	165	45
17	0	67	168	65
18	1	69	143	51
19	1	74	145	53
20	1	72	147	54
21	1	89	137	43
22	0	30	163	68
23	1	77	134	53
24	1	40	149	53
25	1	47	160	48
26	0	84	164	64
27	0	70	180	60
28	0	76	160	60
29	0	73	154	65
30	0	67	165	73
31	1	72	148	40
32	0	79	157	50
33	0	83	152	51
34	0	71	165	62
35	1	83	140	37
36	1	85	150	45
37	0	65	168	75
38	1	29	156	59
39	0	73	157	70
40	0	65	161.4	61.6
41	0	75	163	61
42	0	77	162	51
43	0	46	165	57
44	0	71	166	64
45	1	79	160	75
46	0	73	170	57
47	0	64	185	61
48	0	69	159	62
49	0	54	165	82
50	0	68	167	60
51	0	72	160	24
52	1	78	147	48
53	0	73	167	63
54	0	73	173	57
55	0	84	151	51
56	1	77	148	50
57	0	76	161	71
58	0	69	173	65

59	0	74	160	59
60	0	73	165	60
61	0	71	168	56
62	0	74	158.7	59
63	0	74	161	62
64	0	70	170	80
65	1	81	156	44
66	0	63	170	66
67	1	71	151	51
68	0	62	162	63
69	1	79	143	42
70	0	66	174	49
71	0	66	172	59
72	1	77	144	44
73	0	76	167	48
74	1	69	151	44
75	0	57	163	68
76	0	63	165	70
77	0	60	167	55
78	0	56	172	86
79	1	76	150	45
80	0	56	163	52
81	1	73	149	60
82	0	75	160	47
83	0	77	158	54
84	0	59	178	77.9
85	1	57	153	86

BMI	HT（n:0, y:1）	DL（n:0, y:1）	DM（n:0, y:1）
21.3	1	1	1
19.4	1	1	1
19	1	0	0
21	1	0	1
20.3	1	0	1
23.6	0	0	1
26.2	1	1	0
27.2	1	1	1
18.7	1	0	1
20	1	1	1
21.5	0	0	1
23.5	1	1	1
20.6	1	0	0
25.5	1	1	0
17.1	1	1	0
16.5	0	0	1
23	1	1	1
24.9	1	1	1
25.2	1	1	0
24.9	1	1	0
22.9	1	0	0
25.5	0	0	0
29.5	0	1	0
23.8	1	1	0
18.7	1	1	1
23	1	1	1
18.5	1	0	1
23.4	0	0	0
27.4	1	0	0
26.8	1	1	1
18.2	1	0	0
20.2	1	0	1
22	0	0	0
22.7	1	0	1
18.8	1	0	0
20	1	0	1
26.5	1	0	1
24.2	0	0	1
28.3	1	1	1
23.6	1	0	1
22.9	1	1	1
19.4	1	1	1
20.9	1	0	0
23.2	0	0	1
29.2	1	1	1
19.7	1	1	1
17.8	0	0	1
24.5	1	1	1
30.1	1	1	1
21.5	0	0	0
25	1	1	1
22.2	1	1	0
22.5	0	0	1
19	1	0	0
22.3	0	1	1
22.8	1	1	1
27.3	1	0	0
21.7	0	1	1

23	0	0	0
22	0	1	1
19.8	1	0	0
23.4	1	0	1
23.9	0	0	0
27.6	0	0	1
18	1	0	0
22.8	1	1	0
22.3	1	0	1
14	1	0	0
20.5	1	1	0
16.1	1	0	1
19.9	1	1	0
21.2	0	1	1
17.2	1	0	1
19.2	1	1	0
25.5	1	0	1
25.7	0	1	0
30.4	1	1	1
29	1	0	1
20	1	0	1
19.6	1	1	1
27	1	0	1
18.4	1	0	1
21.6	1	0	1
24.6	1	1	1
36.7	1	1	1

smoking（n:0, ex.:1, curr.:2）	dialysis（n:0, y:1）	collargen disease（n:0, y:1）
1	0	0
1	1	0
0	0	0
2	1	0
1	0	0
0	0	0
0	0	0
2	1	1
0	1	0
0	1	0
1	1	0
2	0	0
1	0	1
1	0	0
1	0	0
2	0	0
0	0	0
0	0	0
0	0	0
0	0	0
0	0	0
2	0	1
0	0	1
2	0	0
2	1	0
1	0	0
1	1	0
2	0	0
1	0	0
2	0	0
0	1	0
1	0	0
0	0	0
1	1	0
0	0	1
0	0	1
1	0	0
0	0	0
1	0	0
1	0	0
0	0	0
2	0	0
0	1	0
1	0	1
0	0	0
2	0	0
1	1	0
2	0	0
0	1	0
1	0	0
1	0	0
0	0	0
1	0	0
0	0	0
0	0	0
0	1	0
0	0	0
1	0	0

1	0	0
1	0	0
1	0	0
0	0	0
1	0	0
0	0	0
0	0	1
1	0	0
1	0	0
2	0	0
0	0	1
1	1	0
0	1	0
0	0	0
2	1	0
1	0	1
2	0	0
1	0	0
2	0	0
0	0	0
2	0	0
2	1	0
0	1	0
0	1	0
0	1	0
1	1	0
0	0	0

Hihger brachial systolic BP	Heart rate	EF (UCG)	Hb
148	76	51	11.7
150	74	69	11.1
111	67	29	16.1
143	63	60	11.9
146	85	47	8.3
166	91	53	15.3
119	67	78	11
134	60	78	11.4
161	71	44	12
151	58	51	11.9
153	55	70	12.3
171	86	78	11.7
146	67	51	10.4
129	47	69	14.3
146	48	73	10.9
122	90	17	12.8
138	64	70	15.2
138	90	65	13.6
100	59	77	12.4
142	81	73	14.4
158	70	79	10.2
120	57	72	15.3
156	94	74	9.9
121	92	80	10.4
154	79	55	7.1
146	63	63	8.3
197	76	50	11
136	72	62	14.9
148	76	56	15.6
136	64	49	11.9
130	60	55	12.4
147	94	70	10.2
120	65	72	12.2
225	67	55	10.9
136	78	53	9.9
124	69	76	9.5
127	69	60	15.8
166	112	66	12.7
120	71	34	14
139	85	60	12.9
200	47	60	13
185	60	49	11.7
137	80	66	13
186	80	83	15.8
155	67	70	11.6
138	57	67	14.6
133	81	67	9.5
132	67	50	16.9
180	66	55	9.7
142	64	62	14.6
134	62	59	12.7
147	65	70	13.2
110	57	53	12.6
146	83	87	13.2
133	53	67	13.5
111	79	67	9.8
135	56	71	15.2
126	81	63	14

154	53	58	12.9
149	79	34	10.6
123	59	60	15.4
154	74	58	9.9
141	85	54	12.8
165	77	49	13.1
163	86	70	11.3
190	76	61	10.1
162	59	47	12.3
104	70	53	15.7
162	60	64	12.3
133	59	72	11
204	54	58	10.8
160	78	34	11.8
162	58	63	8.4
158	54	82	12.4
159	63	54	12.7
151	62	42	13.3
158	61	70	14.3
135	81	68	11.6
131	63	21	12.6
183	80	44	10
118	98	38	12.1
136	81	77	12.4
115	102	29	10
137	76	35	11.7
192	74	64	12.5

CRP	APTT	PT-INR	aspirin(n:0, y:1)	clopidogrel(n:0, y:1)	cirostazol(n:0, y:1)
0.025	33.9	1.02	1	0	0
9.041	32.7	0.98	0	1	0
0.021	33.4	1.02	0	1	0
0.831	31.4	1.06	0	0	0
3.867	35.1	1.03	0	0	0
0.455	37.1	1.04	1	0	0
0.021	27.1	1.02	0	0	0
0.903	42.9	1.19	0	0	0
4.097	31.4	1.02	1	0	0
0.036	38.7	1.19	1	0	0
1.761	30.9	1.02	0	0	1
0.091	33.7	1.01	1	0	1
2.432	29.1	1.02	0	0	1
0.092	31	0.95	1	0	0
0.014	29.1	1.07	1	0	0
0.19	31.3	1.03	0	0	0
0.137	28.2	1.01	0	0	0
0.019	29.9	0.98	0	0	0
0.039	33.5	0.93	1	0	0
0.219	47.9	1.16	1	0	0
1.182	40.7	1.21	1	0	0
0.625	26.3	0.99	0	0	1
1.664	39.3	1.06	0	0	0
0.213	36.1	1.07	0	0	1
24.507	35.3	1.24	0	0	0
0.324	47.1	2.75	0	0	1
8.399	37.5	1.2	1	0	0
0.029	27.4	0.9	0	0	0
0.06	34.1	1.02	1	0	0
0.729	30.6	1.05	1	0	0
0.432	42	1.47	1	0	0
2.082	36	1.19	1	0	1
1.194	63.4	1.43	0	0	0
0.472	32.4	1.14	1	0	0
0.787	30.7	1	0	0	0
2.642	26.7	1.01	0	0	0
0.125	28.5	1.01	0	0	0
2.301	33.5	1.04	0	0	0
0.171	42.3	2.17	1	1	0
0.421	27.3	0.97	1	0	1
0.019	27.8	0.96	1	1	0
0.016	26.4	0.95	1	1	0
0.159	30.8	1.12	1	1	0
0.1	32.7	1.06	0	0	0
0.42	33.6	1.08	1	1	0
0.037	28.2	0.96	1	1	0
5.868	36	1.18	0	1	0
0.349	31.2	1.01	1	0	0
0.769	31.6	1.12	0	1	0
0.122	42	1.62	1	0	0
0.034	31.4	0.98	1	1	0
1.658	30.6	1.11	0	1	0
0.044	30	0.97	1	0	0
0.014	31.6	1.08	0	0	0
0.01	31.6	0.96	0	0	0
8.68	50.4	3.93	0	0	0
0.307	27.9	0.92	0	0	0
0.024	29.6	1.05	0	1	0

0.08	29.2	1.07	0	0	0
2.934	30.3	1.17	0	1	0
0.3	28.8	1.06	0	0	0
1.174	32	0.96	0	0	0
1.315	29.5	0.98	0	0	0
0.165	32.7	0.97	0	0	1
2.386	37.8	1.08	0	0	1
5.879	29.3	1.17	1	1	0
0.06	31.4	2.66	1	0	0
0.022	30.5	1.01	1	0	0
0.201	30.8	1.01	1	0	0
8.116	42.1	1.57	1	0	0
0.079	31.1	1.08	1	0	0
0.021	22.9	1.11	1	1	0
1.553	27.6	1.04	1	0	1
0.703	26.5	1.07	0	0	1
0.097	29.9	1.02	0	1	1
0.025	29.6	0.91	1	0	1
0.743	31.1	1.04	1	0	0
3.458	27.4	1.26	0	0	0
0.249	56.2	1.12	1	0	0
12.796	34	1.12	0	1	1
0.817	47.8	2.72	1	1	1
0.546	32.8	1.11	1	0	1
5.623	43.5	1.44	1	0	0
0.142	25.3	1.06	1	1	0
0.608	26.5	1.03	0	0	0

warfarin（n:0, y:1）	statin（n:0, y:1）	ACEI（n:0, y:1）	iliac-femoral CTO	Rutherford grade
0	1	0	0	3
0	0	0	0	6
0	0	0	0	1
0	0	0	0	3
0	0	0	0	6
0	0	0	1	2
0	1	1	1	3
0	0	0	0	5
0	0	0	0	5
0	1	0	1	6
0	0	0	0	5
0	1	0	1	3
0	0	0	0	6
0	1	0	0	1
0	1	0	1	1
0	0	0	1	1
0	1	0	0	2
0	0	0	1	1
0	1	0	1	3
0	1	0	1	2
0	0	0	1	6
1	0	0	1	2
0	0	0	1	6
0	1	0	1	2
0	0	0	0	6
1	1	0	0	5
0	0	0	1	6
0	0	0	0	2
0	0	0	1	1
0	1	0	1	3
1	0	0	0	6
0	0	1	1	5
1	0	0	0	3
0	0	0	0	4
0	0	0	0	6
0	0	0	0	5
0	0	0	0	1
0	0	0	1	6
1	0	0	0	3
0	0	0	1	3
0	1	0	1	1
0	1	0	1	3
0	0	0	0	4
0	0	0	0	3
0	0	0	0	5
0	1	0	0	5
0	0	0	0	5
0	1	0	1	3
0	0	0	0	6
1	0	0	0	3
0	1	0	1	3
0	1	1	0	2
0	0	0	0	3
0	0	0	0	2
0	0	0	0	3
1	0	0	0	5
0	0	0	0	3
0	1	0	0	2

0	0	0	1	3
0	1	0	0	6
1	1	0	0	3
0	0	0	1	6
0	0	0	1	3
0	0	0	0	5
0	0	0	0	6
0	0	0	1	2
1	0	1	0	2
0	1	1	1	2
0	1	0	0	5
1	0	0	0	6
0	1	0	0	4
1	1	0	0	5
0	0	0	0	3
0	1	0	0	3
0	1	0	1	3
0	1	1	0	2
0	0	0	0	6
0	0	0	0	6
0	0	0	1	4
0	1	1	1	6
0	0	0	0	5
0	0	0	0	6
0	0	0	0	6
0	1	0	1	5
0	0	0	1	6

diseased ankle systolic BP	diseased ankle diastolic BP	diseased ankle pulse pressure
132	74	58
62	47	15
79	57	22
126	72	54
87	25	62
146	90	56
68	48	20
135	64	71
143	60	83
107	38	69
122	82	40
95	58	37
154	54	90
110	66	44
102	78	24
79	60	19
123	86	37
102	74	28
69	47	22
88	71	17
148	58	90
64	53	11
89	63	26
51	35	16
100	36	64
111	42	69
90	60	30
81	65	16
115	82	33
92	57	35
112	70	42
51	24	27
78	67	11
146	69	77
85	39	46
82	49	33
87	60	27
97	66	31
78	63	15
136	64	72
112	87	25
105	75	30
60	38	22
136	89	47
144	72	72
136	64	68
96	51	45
88	41	47
136	78	58
75	68	7
87	67	20
112	70	42
74	51	23
95	68	27
86	55	31
83	33	50
94	56	38
105	68	37

77	52	25
97	56	41
110	79	31
90	61	29
87	57	30
84	62	22
162	82	80
102	83	19
94	60	34
61	43	18
156	64	92
99	31	68
139	86	53
84	75	9
70	52	18
87	65	22
102	82	20
117	72	45
82	65	17
126	87	39
54	44	10
92	34	58
62	37	25
110	46	64
100	72	28
107	45	62
92	78	14

diseased ankle mean arterial pressure	ABI	AHI
94	0.711711712	21.05714286
50	0.571428571	22.71186441
70	1.054794521	59.11764706
93	0.852713178	24.91566265
58	0.698630137	12.8
111	0.69	21.63333333
59	0.61971831	18.60810811
89	0.936708861	50
100	0.533333333	11.19642857
81	0.570512821	33.94444444
105	0.421487603	36.8
85	0.595588235	16.69565217
102	0.777027027	25.59183673
83	0.861538462	25.71428571
90	0.65	10.21428571
72	0.625	67.69811321
104	0.437956204	42.92682927
84	0.528169014	6.309859155
60	0.761904762	32.89156627
74	0.650684932	28.36708861
126	0.696296296	32.24242424
56	0.5	19.7761194
72	0.894308943	20.5505618
40	0.617021277	35.91549296
73	0.993865031	56.39344262
73	0.536842105	16.59770115
75	0.586538462	26.80851064
69	0.962962963	51.11111111
98	0.681372549	26.25688073
69	0.550632911	14.66666667
98	0.774834437	30.32608696
37	0.891891892	46.89361702
70	0.413333333	22.2
108	0.881118881	36.58064516
53	0.595890411	90.86206897
68	0.879518072	45.90990991
71	1.007462687	47.86516854
76	0.888198758	58.93
66	0.708609272	49.40740741
87	0.797385621	20.95238095
93	0.555555556	37.43529412
90	0.647540984	23.75
41	0.891304348	22.76923077
105	0.739130435	30
89	0.649350649	69.26027397
83	0.760273973	59.54794521
97	0.456852792	30.4
53	0.676470588	32.46376812
100	0.346938776	68.59459459
71	0.648888889	47.76851852
77	0.661290323	33.48529412
83	0.68503937	26.23943662
63	0.584337349	45.68421053
79	0.65	16.13636364
70	0.978417266	70.34482759
85	0.56	12.6344086
66	0.567567568	20
82	0.731182796	35.80952381

67	0.929032258	54.20224719
77	0.985507246	46.69879518
89	0.721804511	37.57731959
72	0.666666667	59.41509434
71	0.755555556	38.28
73	0.649253731	16.1038961
122	0.672727273	20.80952381
87	0.646616541	23.47142857
80	0.747747748	46.47058824
47	0.833333333	36.54878049
108	0.651006711	42.06493506
66	0.584415584	29.80555556
109	0.509090909	23.20547945
79	0.580246914	25.075
57	0.744360902	60.78787879
81	0.525	8.886075949
92	0.432098765	18.31578947
92	0.641509434	13.69565217
69	0.518987342	15.02898551
105	0.933333333	30.08571429
54	0.41221374	11.66666667
65	0.50273224	71.38461538
44	0.525423729	55.68181818
86	0.808823529	60.27906977
81	0.869565217	35.25925926
84	0.781021898	56.0952381
82	0.479166667	12.63414634
